# Hyperglycemia 72 h before necrotizing enterocolitis onset and mortality among premature and term infants in Kenya

**DOI:** 10.7717/peerj.21601

**Published:** 2026-08-25

**Authors:** Marion Muhia, Roseline Ochieng, Angela Migowa

**Affiliations:** 1 Pediatrics Department, Thika Level V Hospital, Thika, Kenya; 2 Department of Pediatrics and Child Health, Aga Khan University, Nairobi, Kenya

**Keywords:** Hyperglycemia, Necrotizing enterocolitis, Blood glucose, Mortality, Infant, Neonate, Preterm, Incidence, Noninvasive ventilation, Surgery

## Abstract

**Background:**

Necrotizing enterocolitis (NEC) is a common complication among preterm infants but can also occur in term babies. A potential relationship between the onset of NEC and hyperglycemia has been suggested, but some studies report no association, necessitating further research to clarify the link between the two conditions. This study aimed to determine the proportion of NEC cases with hyperglycemia 72 h before its onset in preterm and term infants, identify clinical factors associated with hyperglycemia in these infants, and investigate the relationship between hyperglycemia and in-hospital mortality.

**Methods:**

A retrospective cohort study was conducted at the Aga Khan University Hospital in Nairobi, Kenya. The participants included preterm and term infants treated for NEC at the hospital between January 1, 2014, and December 31, 2024. Patient data were retrieved from the electronic health records and paper files. Random blood sugar records 72 h before the diagnosis of NEC were assessed. Hyperglycemia was diagnosed based on a minimum of two blood glucose measurements exceeding 8.3 mmol/l (150 mg/dl). The study outcomes were proportion of NEC cases with hyperglycemia 72 h before its onset and in-hospital mortality. A generalized linear regression model with binomial probability distribution was used to identify the factors associated with hyperglycemia incidence in neonates diagnosed with NEC.

**Results:**

The proportion of NEC cases with hyperglycemia 72 h before its onset in preterm and term infants was 46.3%. Among the participants, 20.9% (*n* = 14) died before discharge, with no significant difference between those with (*n* = 8, 25.8%) and without (*n* = 6, 16.7%) hyperglycemia (*p* = 0.36). On multivariate analysis, the use of synchronized intermittent mandatory ventilation/non-invasive ventilation versus oxygen/room air was associated with an increased risk of hyperglycemia diagnosis. NEC requiring surgical intervention showed a more than three-fold risk of mortality compared with NEC treated with medical/supportive care only.

**Conclusion:**

The results show that nearly half (46.3%) of the preterm and term infants with NEC were diagnosed with hyperglycemia 72 h before its onset. The diagnosis of hyperglycemia was not associated with in-hospital mortality. These findings should be validated in larger, adequately powered studies before clinical application.

## Introduction

Necrotizing enterocolitis (NEC) is a severe medical condition that predominantly impacts premature babies, although it can also manifest in full-term infants. Globally, the incidence of NEC ranges from 0.3 to 2.4 cases per 1,000 live births (Ginglen & Butki, 2025). Approximately 70% of these cases involve premature infants delivered before 36 weeks of gestation. The underlying mechanism involves inflammation within the intestines, leading to invasion of bacteria into the intestinal mucosa. Over time, this process results in damage to cells and necrosis affecting both the colon and small intestine (Kosloske & Musemeche, 1989). While the precise cause of NEC remains insufficiently understood, various factors such as prematurity, formula feeding, intestinal bacterial colonization, and a weakened immune system are posited to collectively contribute to its development (Kosloske & Musemeche, 1989).

In neonates with NEC admitted to the intensive care unit (ICU), hyperglycemia is prevalent and linked to heightened late mortality rates and prolonged stays in the ICU (Hall et al., 2004). The estimated incidence of neonatal hyperglycemia is approximately 45–80% (Parappil et al., 2022). Research on the association between hyperglycemia and the risk of NEC is contradictory, with some studies reporting an increased risk of NEC in premature or term infants who experience hyperglycemia (Hall et al., 2004; Gul et al., 2017) and others reporting no association (El-Shimi, El-Saoud & Ismail, 2022). The exact mechanisms linking hyperglycemia to NEC are not fully understood; however, it is hypothesized that hyperglycemia induces excessive production of free radicals, which cause oxidative stress and damage to cells, including the intestinal barrier, potentially raising the risk of NEC (Thaiss et al., 2018; Guo et al., 2020). Severe and prolonged hyperglycemia are linked to poor neonatal outcomes, including NEC, owing to the longer duration of metabolic dysregulation (Alsayigh et al., 2026). Glucagon-like peptide-2 has been shown to exert anti-inflammatory effects in a rat model of NEC by suppressing the production of the tumor necrosis factor-alpha, a key mediator of inflammation (Nakame et al., 2016). While there is some evidence suggesting an association, researchers emphasize the need for further investigation to establish a clear and causative link.

A previous study reported an increase in blood glucose levels 1–3 days before the onset of NEC in preterm infants (Hällström et al., 2006). Most cases of NEC have also been shown to evolve over a period of 24–48 h from a prodromal phase to advanced clinical manifestations (Pammi, De Plaen & Maheshwari, 2020). Therefore, it is important to determine the number of preterm and term infants who develop hyperglycemia 72 h before NEC onset and explore whether the hyperglycemic status is associated with poor outcomes. Continuous glucose monitoring is a technique that measures glucose levels in real-time and has been shown to capture glycemic fluctuations in neonates (Iglesias Platas et al., 2009; Musso et al., 2022). As such, a sudden rise in blood glucose levels, which corresponds to advanced NEC stage, need for surgical intervention, and high risk of mortality, can be detected in a timely manner (Hall et al., 2004). A high index of suspicion for possible NEC in premature and term infants presenting with hyperglycemia will enable the health worker to be more vigilant with history taking, physical examination, and laboratory result assessment. Early diagnosis of NEC may help reduce morbidity and mortality and improve neonatal health indicators.

This study aimed to determine the proportion of NEC cases with hyperglycemia 72 h before its onset among preterm and term infants, identify clinical factors associated with hyperglycemia in these infants, and investigate the relationship between hyperglycemia and in-hospital mortality.

## Materials & Methods

### Study design

This retrospective cross-sectional study collected data for preterm and term infants admitted to neonatal intensive care unit or neonatal high dependency unit with a diagnosis of NEC between January 1, 2014, and December 31, 2024. The study was conducted at the Aga Khan hospital in Nairobi. The research was approved by the Aga Khan University Hospital-Institution Scientific Research and Ethics Committee (approval number: 2024/ISERC-43 (v5)), who also granted a consent waiver. Authors had access to patient-identifying information, and thus patient data were deidentified through codes. Confidentiality was strictly upheld throughout data collection, analysis, and dissemination of results.

### Study participants

The study involved all preterm and term infants meeting the enrollment criteria during the study period. The inclusion criterion was a diagnosis of NEC in a preterm or term infant. The exclusion criterion was a diagnosis of congenital anomalies.

No sample size was estimated for this study. All available eligible preterm/term neonates during study period were included in the study (*N* = 67). The available sample of 67 neonates yielded a 95% confidence interval with a half-width of approximately 12%, indicating reasonable precision for estimating the proportion of NEC cases with hyperglycemia

### Data collection

The author (MWM) extracted patient data from the electronic health records (EHR)—Care2000 and Meditech systems—from 19 March 2025 to 19 August 2025. The Care2000 system was used in the hospital up to November 2023, after which it was replaced by the Meditech system. In addition, more data were extracted from the paper files. International Classification of Diseases (ICD) codes for NEC were obtained and used to identify patient records that met the eligibility criteria. Additional data included random blood sugar (RBS) values in mmol/l recorded 72 h before the diagnosis of NEC, gestational age at birth (weeks), sex, birth weight (grams), mode of delivery (categorized into three groups: vaginal delivery, emergency cesarian section, or elective cesarian section), and maternal risk factors (diabetes [ICD-10 codes: E08–13], hypertension [I10–I15], chorioamnionitis [O41.12]). Further data included postnatal age (days), mode and type of feeding, mode of ventilation, and associated comorbidities (sepsis [A40–A41], respiratory distress syndrome [P22], patent ductus arteriosus [Q25.0]) at the time of diagnosis.

MWM used the Research Electronic Data Capture (REDCap) for data abstraction. The author manually navigated through the system and reviewed patients’ clinical notes, as well as demographic and laboratory characteristics. The variables of interest were manually extracted and entered into the REDCap for data abstraction. Data missing from the EHR systems were manually extracted from the patients’ files in the archives.

### Study outcomes

The primary outcome was the proportion of NEC cases with hyperglycemia 72 h before its onset among preterm and term infants. The modified Bell staging criteria were used to categorize the diagnosis of NEC (Walsh & Kliegman, 1986). The stages include 1A suspected; 1B suspected; IIA definite, mildly ill; IIB definite, moderately ill; IIIA advanced, severely ill, intact bowel; and IIIB advanced, severely ill, perforated bowel. NEC stages were abstracted from the medical records, and no independent adjudication was performed. Hyperglycemia was defined by a minimum of two (Kosloske & Musemeche, 1989) RBS measurements exceeding 8.3 mmol/l (150 mg/dl). The severity of neonatal hyperglycemia was categorized as mild for blood glucose level >8.3–10 mmol/L (>150–180 mg/dl), moderate for blood glucose level >10–15 mmol/L (>180–270 mg/dl), and severe for blood glucose level >15 mmol/L (>270 mg/dl) (Parappil et al., 2022). The secondary outcome was in-hospital death or recovery of the preterm and term infants with hyperglycemia diagnosed with NEC.

### Data analysis

All categorical variables are reported as frequencies with percentage while continuous variables are reported as median (interquartile range—IQR) because they were skewed. NEC cases with hyperglycemia 72 h before its onset and in-hospital deaths are reported as a proportion of the study participants. Proportions of hyperglycemia and death between groups were compared using the chi-square test of association.

To identify factors associated with the occurrence of hyperglycemia 72 h before the diagnosis of NEC, a generalized linear regression model (GLM) with binomial probability distribution (for dependent variable) and logit link were fixed because the prevalence of the dependent variable, *i.e.,* incidence of hyperglycemia, was >10% (not a rare event) (Zou, 2004). The variables considered for the GLM included gestational age at birth (Maayan-Metzger et al., 2004), sex (Maayan-Metzger et al., 2004), birthweight (grams) (Beardsall et al., 2010; Banik et al., 2014), mode of delivery categorized into three groups (vaginal delivery, emergency, or cesarian section) (Maayan-Metzger et al., 2004), maternal risk factors (diabetes, hypertension, chorioamnionitis) (Essex et al., 2024), postnatal age (days) (Alsayigh et al., 2026), mode and type of feeding (Maayan-Metzger et al., 2004), mode of ventilation (Alsayigh et al., 2026), and comorbidities (sepsis, respiratory distress syndrome, patent ductus arteriosus) (Stout et al., 2008) at the time of diagnosis.

In the univariate analysis, GLMs were performed for each independent variable and crude risk ratio (CRR) with 95% confidence intervals (CI) were reported. All independent variables with a *P*-value < 0.2 in the univariate analysis were retained in the multivariable model. Similarly, GLMs were used to identify factors associated with inpatient deaths, and risk ratios were reported as measures of effects. All statistical analyses were conducted using STATA version 17.0 (College Station, TX: StataCorp LLC).

## Results

### Participant characteristics

The study included a total of 67 participants (female, *n* = 35; median gestational age, 35 weeks). Of the participants, 7 (10.5%) were extreme preterm, 41 (61.2%) preterm, and 19 (28.3%) term. The median (IQR) birth weight was 1,815 (1,160–2,800) g, and 11 (16.4%) and 35 (52.2%) were born with extreme low birth weight and low birth weight, respectively. Majority (*n* = 64, 95.5%) had Appearance, Pulse, Grimace, Activity, Respiration (APGAR) scores ≥7 at 5 min after birth. Emergency cesarian section (*n* = 47, 70.2%) was the most frequent mode of delivery. The median (IQR) post-natal age at NEC diagnosis was 7 (4–11) days. Over 80% (*n* = 57, 85.1%) were diagnosed with NEC stage 2A (confirmed). The most frequent maternal medical histories were gestational diabetes (*n* = 12, 17.9%), preterm prelabor rupture of membranes (PPROM) > 18 h (*n* = 9, 13.4%), and gestational hypertension (*n* = 7, 10.5%). A total of 31 (46.3%) participants had a history of antenatal steroid administration (Table 1).

**Table 1:** Participant characteristics.

**Characteristics (*N* = 67)**	**N (%)**
Sex	
Male	32 (47.8)
Female	35 (52.2)
Gestational age in weeks; median (IQR)	32 (30–37)
Extreme preterm (<28 weeks)	7 (10.5)
Preterm (28–36 weeks)	41 (61.2)
Term (≥37 weeks)	19 (28.4)
Birth weight (grams); median (IQR)	1,815 (1,160–2,800)
Extreme low birth weight (<1,000 grams)	11 (16.4)
Low birth weight (1,000–2,499 grams)	35 (52.2)
Normal birth weight (≥2,500 grams)	21 (31.3)
APGAR score at 5 min	
<7	3 (4.5)
≥7	64 (95.5)
Mode of delivery	
Elective cesarian section	8 (11.9)
Vaginal	9 (13.4)
Emergency cesarian section	47 (70.2)
Vacuum extraction	3 (4.5)
**Patient medical history**	
Post-natal age at NEC diagnosis; median (IQR)	7 (4–11)
NEC staging	
Stage 1A (suspected)	1 (1.5)
Stage 2A (Confirmed)	57 (85.1)
Stage 2B (Confirmed)	1 (1.5)
Stage 3A (Surgical)	3 (4.5)
Stage 3B (Surgical)	5 (7.5)
**Maternal history**	
No complication	38 (56.7)
PPROM >18 h	9 (13.4)
Pregestational hypertension	3 (4.5)
Pregestational diabetes	2 (2.9)
Gestational diabetes	12 (17.9)
Gestational hypertension	7 (10.5)
History of antenatal steroid administration	
No	36 (53.7)
Yes	31 (46.3)

**Notes.**

All results are N (%) unless they are specified.

Abbreviations APGAR Appearance, Pulse, Grimace, Activity, Respiration IQR interquartile range NEC necrotizing enterocolitis PPROM preterm prelabor rupture of membranes

### Prevalence of hyperglycemia

Overall, 31/67 patients with NEC were diagnosed with hyperglycemia 72 h before NEC onset. The proportions with and without hyperglycemia were not significantly different (*P* = 0.39). Among the 31 hyperglycemia cases, the median (IQR) RBS highest reading was 11.6 (10.1–16.1) mmol/l. More than three quarters (*n* = 24, 77.4%) of the 31 hyperglycemia cases had either moderate or severe hyperglycemia. Over three quarters (*n* = 51, 76%) were on caffeine citrate medication, 2 (3.0%) on Inotropes, and 34 (50.8%) on intravenous dextrose. Overall, 34 (50.8%) did not require ventilation (were breathing on room air), 6 (9.0%) were on oxygen supplementation, 2 (3.0%) on non-invasive ventilation (NIV), and 25 (37.3%) on synchronized intermittent mandatory ventilation (SIMV). The leading comorbidities were respiratory distress syndrome (RDS) (*n* = 45, 67.2%), sepsis (*n* = 25, 37.3%), neonatal jaundice (NNJ) (*n* = 24, 35.8%), and coagulopathy (*n* = 21, 31.3%). One-fifth (*n* = 14, 20.9%) required surgical intervention to treat NEC. The proportion of patients with NEC stage 3 was 9.68% and 13.9% among those with and without hyperglycemia (*P* = 0.6) (Table 2).

**Table 2:** Diagnosis of hyperglycemia and its characteristics.

**Characteristics**	**Hyperglycemia**		**Overall**
	**No (*n* = 36)**	**Yes (*n* = 31)**	***N* = 67**
RBS highest reading in mmol/l; median (IQR)	5.6 (5.0–6.2)	11.6 (10.1–16.1)	7.1 (5.6–11.4)
Severity of hyperglycemia			
Normal	36 (100)	2 (6.5)	38 (56.7)
Mild—blood glucose >8.3 mmol/l	0	5 (16.1)	5 (7.5)
Moderate—blood glucose > 10 mmol/l	0	13 (41.9)	13 (19.4)
Severe—blood glucose > 15 mmol/l	0	11 (35.5)	11 (16.4)
Duration of hyperglycemia in hours; median (IQR)	–	3 (2–14)	3 (2–14)
Mode of feeding before NEC diagnosis			
Formula only	5 (13.9)	3 (9.7)	8 (11.9)
Mothers milk only	12 (33.3)	17 (54.8)	29 (43.3)
EBM & formula combined	17 (47.2)	9 (29.0)	26 (38.8)
EBM, formula, and TPN	2 (5.6)	2 (6.5)	4 (6.0)
NEC staging			
Stages 1 and 2	31 (86.1)	28 (90.3)	59 (88.1)
Stage 3	5 (13.9)	3 (9.7)	8 (11.9)
Current medication^*^			
None	7 (19.4)	0	7 (10.5)
Caffeine citrate	28 (77.8)	24 (77.4)	52 (77.6)
Inotropes	0	2 (6.5)	2 (3.0)
Intravenous dextrose	7 (19.4)	27 (87.1)	34 (50.8)
GIR rate for Intravenous dextrose only: median (IQR)	–	5.6 (4–8)	5.6 (4–8)
Type of ventilation			
SIMV	7 (19.4)	18 (58.1)	25 (37.3)
NIV	2 (5.6)	0	2 (3.0)
Oxygen only	3 (8.3)	3 (9.7)	6 (9.0)
Room air	24 (66.7)	10 (32.3)	34 (50.8)
Days on ventilation (*n* = 33); median (IQR)	3 (2–4.5)	3 (2–4)	3 (2–4)
Comorbidities			
RDS	21 (58.3)	24 (77.4)	45 (67.2)
Sepsis	12 (33.3)	13 (41.9)	25 (37.3)
PDA	1 (2.8)	5 (16.1)	6 (9.0)
Coagulopathy	8 (22.2)	13 (41.9)	21 (31.3)
IVH	2 (5.6)	6 (19.4)	8 (11.9)
Pneumothorax	1 (2.8)	2 (6.5)	3 (4.5)
NNJ	11 (30.6)	13 (41.9)	24 (35.8)
Anemia	4 (11.1)	2 (6.5)	6 (9.0)
Viral hepatitis	1 (2.8)	0	1 (1.5)
Perinatal asphyxia	3 (8.3)	1 (3.2)	4 (6.0)
Thrombocytopenia	3 (8.3)	2 (6.5)	5 (7.5)
IUGR	1 (2.8)	0	1 (1.5)
Neonatal seizures	0	3 (9.7)	3 (4.5)
BPD	1 (2.8)	2 (6.5)	3 (4.5)
ROP	0	2 (6.5)	2 (3.0)
Apnea of prematurity	1 (2.8)	1 (3.2)	2 (3.0)
NEC treatment			
Medical/supportive care only	30 (83.3)	23 (74.2)	53 (79.1)
Required surgical intervention	6 (16.7)	8 (25.8)	14 (20.9)

**Notes.**

* A patient can get more than one medication. All results are N (%) unless specified otherwise.

Abbreviations BPD bronchopulmonary dysplasia EBM expressed breast milk GIR glucose infusion rate IQR interquartile range IUGR intrauterine growth restriction IVH intraventricular hemorrhage NEC necrotizing enterocolitis NIV non-invasive ventilation NNJ neonatal jaundice PDA patent ductus arteriosus RBS random blood sugar ROP Retinopathy of prematurity RSS respiratory distress syndrome SIMV synchronized intermittent mandatory ventilation TPN total parental nutrition

### Factors associated with hyperglycemia in NEC cases

In the univariate analysis, SIMV/NIV *versus* room air was associated with more than two-fold risk of hyperglycemia diagnosis (CRR 2.27, 95% CI [1.26–4.07], *P* = 0.006). Comorbidity of intraventricular hemorrhage (IVH) was associated with a 77% higher risk of hyperglycemia diagnosis (CRR 1.77, 95% CI [1.08–2.91], *P* = 0.03). Gestation age of 28–36 weeks was negatively associated with hyperglycemia diagnosis (CRR 0.49, 95% CI [0.33–0.71], *P* < 0.001) compared with term birth (gestation age ≥37 weeks) (Table 3).

**Table 3:** Univariate analysis of factors associated with incidence of hyperglycemia.

			**Univariate analysis**		**Multivariate analysis**	
**Factor**	**Hyperglycemia**		**Crude risk Ratio (CRR)** **95% CI**	***P*-** **value**	**Adjusted risk** **Ratio (aRR) 95% CI**	***P*-value**
	No (*n* = 36)	Yes (*n* = 31)				
Sex						
Male	18 (50.0)	14 (45.2)	Reference			
Female	18 (50.0)	17 (54.8)	1.11 (0.66–1.87)	0.69		
Gestation age (weeks)						
≥37 weeks (term)	10 (27.8)	9 (29.0)	Reference		Reference	
28–36 weeks	25 (69.4)	16 (51.6)	0.49 (0.33–0.71)	**<0.001**	1.44 (0.34–6.03)	0.62
<28 weeks	1 (2.8)	6 (19.4)	¶		1.37 (0.12–15.2)	0.80
Birth weight (grams)						
≥2,500 (normal birth weight)	10 (27.8)	11 (35.5)	Reference		Reference	
1,000–2,499 grams	23 (63.9)	12 (38.7)	0.65 (0.35–1.21)	0.18	0.48 (0.12–1.96)	0.30
<1,000 grams	3 (8.3)	8 (25.8)	1.39 (0.80–2.40)	0.24	0.73 (0.09–6.21)	0.78
APGAR within 5 min						
≥7	35 (97.2)	29 (93.6)	Reference			
<7	1 (2.8)	2 (6.5)	1.47 (0.63–3.42)	0.37		
Mode of delivery						
Vaginal	3 (8.3)	6 (19.4)	Reference		Reference	
Elective/Emergency C-S/ Vacuum Extraction	33 (91.7)	25 (80.6)	0.65 (0.37–1.12)	0.12	0.60 (0.21–1.66)	0.32
Post-natal age at NEC diagnosis (log)	–	–	1.07 (0.71–1.60)	0.75		
NEC staging						
Stages 1 and 2	31 (86.1)	28 (90.3)	Reference			
Stage 3	5 (13.9)	3 (9.7)	0.79 (0.31–2.01)	0.62		
Maternal history^*^						
No complication	22 (61.1)	16 (51.6)	0.81 (0.49–1.36)	0.43		
PPROM > 18 h	5 (13.9)	4 (12.9)	0.95 (0.44–2.08)	0.91		
Pregestational hypertension	2 (5.6)	1 (3.2)	0.71 (0.14–3.60)	0.68		
Gestational diabetes	5 (13.9)	7 (22.6)	1.34 (0.76–2.35)	0.31		
Gestational hypertension	3 (8.3)	4 (12.9)	1.27 (0.63–2.56)	0.50		
History of antenatal steroid administration						
No	19 (52.8)	17 (54.8)	Reference			
Yes	17 (47.2)	14 (45.2)	0.96 (0.57–1.61)	0.87		
Mode of feeding before NEC diagnosis						
Mothers milk only	12 (33.3)	17 (54.8)	Reference		Reference	
Formula only	5 (13.9)	3 (9.7)	0.64 (0.25–1.65)	0.35	0.78 (0.20–3.05)	0.73
EBM & formula combined	17 (47.2)	9 (29.0)	0.59 (0.32–1.09)	0.09	0.65 (0.28–1.54)	0.33
EBM, formula, and TPN	2 (5.6)	2 (6.5)	0.85 (0.31–2.28)	0.76	1.24 (0.27–5.79)	0.78
Type of ventilation at diagnosis						
Room air	24 (66.7)	10 (32.3)	Reference		Reference	
Oxygen only	3 (8.3)	3 (9.7)	1.70 (0.65–4.42)	0.28	1.71 (0.43–6.70)	0.44
SIMV/NIV	9 (25.0)	18 (58.10)	2.27 (1.26–4.07)	**0.006**	2.32 (1.05–5.14)	**0.04**
Comorbidities^*^						
RDS	21 (58.3)	24 (77.4)	1.68 (0.86–2.28)	0.13	1.41 (0.53–3.75)	0.49
Sepsis	12 (33.3)	13 (41.9)	1.21 (0.73–2.03)	0.46		
Coagulopathy	8 (22.2)	13 (41.9)	1.58 (0.97–2.59)	0.07	1.33 (0.53–3.29	0.54
IVH	2 (5.6)	6 (19.4)	1.77 (1.08–2.91)	**0.03**	1.80 (0.59–5.49)	0.30
NNJ	11 (30.6)	13 (41.9)	1.29 (0.78–2.15)	0.32		
NEC treatment						
Medical/supportive care only	30 (83.3)	23 (74.2)	Reference			
Required surgical intervention	6 (16.7)	8 (25.8)	1.32 (0.76–2.28)	0.33		

**Notes.**

Risk Ratios were obtained from Generalized Linear Models with binomial probability distribution and logit link. All independent variables with a *P*-value < 0.2 in the univariate analysis were retained in the multivariable model.

* The reference group is “not present” for each medical history or comorbidity. ¶, insufficient numbers.

Abbreviations APGAR Appearance, Pulse, Grimace, Activity, Respiration CI confidence interval C-S cesarean section EBM expressed breast milk IVH intraventricular hemorrhage NEC necrotizing enterocolitis NIV non-invasive ventilation NNJ neonatal jaundice PPROM preterm prelabor rupture of membranes RDS respiratory distress

Bolded values indicate statistical significance (*P* < 0.05).

However, in the multivariable model, the effects of IVH comorbidity and gestation age attenuated. SIMV/NIV *versus* room air was associated with more than two-fold risk of hyperglycemia diagnosis (adjusted risk ratio (aRR) 2.32, 95% CI [1.05–5.14], *P* = 0.04) (Table 3).

### Factors associated with in-hospital mortality

Overall, 14/67 (20.9%) participants died before discharge from the hospital. Mortality rate was not significantly different between the patients with (*n* = 8, 25.8%) and without (*n* = 6, 16.7%) hyperglycemia (*P* = 0.36). Similarly, the mortality rate was not significantly different among the term (*n* = 2, 10.5%), preterm (*n* = 9, 22.0%), and extreme preterm (*n* = 3, 42.9%) neonates (*P* = 0.18). However, the mortality rate was significantly higher among the extreme low birth weight (LBW) (*n* = 5, 45.5%) than among the LBW (*n* = 8, 22.9%) and normal birth weight (*n* = 1, 4.8%) neonates (*P* = 0.03).

In the univariate analysis, extreme low birth weight was associated with more than 9-fold risk of mortality compared with normal birth weight (CRR 9.55, 95% CI [1.27–71.9], *P* = 0.03). Maternal history of gestational hypertension was associated with an over 3-fold risk of mortality (CRR 3.43, 95% CI [1.46–8.07], *P* = 0.005). History of antenatal steroid administration had a CRR of 2.90 (95% CI [1.01–8.34], *P* = 0.04) for mortality. Compared with breathing in room air, SIMV/NIV was associated with an over 7-fold risk of mortality (CRR 7.56, 95% CI [1.85–30.9], *P* = 0.005). Comorbidity of coagulopathy (CRR 5.48, 95% CI [1.94–15.5]) and NNJ (CRR 0.14, 95% CI [0.02–0.99]) were significantly associated with mortality. NEC requiring surgical intervention had a CRR of 3.79 (95% CI [1.59–9.00]) for mortality compared with medical/supportive care only (Table 4).

**Table 4:** Multivariable analysis of factors associated with incidence of hyperglycemia.

**Factor**	**Adjusted risk** **Ratio (aRR) 95% CI**	***P*-value**
Sex		
Male	Reference	
Female	1.01 (0.48–2.13)	0.97
Gestation age (weeks)		
≥37 weeks (term)	Reference	
28–36 weeks	1.44 (0.34–6.03)	0.62
<28 weeks	1.37 (0.12–15.2)	0.80
Birth weight (grams)		
≥2,500 (normal birth weight)	Reference	
1,000–2,499 grams	0.48 (0.12–1.96)	0.30
<1,000 grams	0.73 (0.09–6.21)	0.78
Mode of delivery		
Vaginal	Reference	
Elective/Emergency C-S/Vacuum Extraction	0.60 (0.21–1.66)	0.32
Mode of feeding before NEC diagnosis		
Mothers milk only	Reference	
Formula only	0.78 (0.20–3.05)	0.73
EBM & formula combined	0.65 (0.28–1.54)	0.33
EBM, formula, and TPN	1.24 (0.27–5.79)	0.78
Type of ventilation at diagnosis		
Room air	Reference	
Oxygen only	1.71 (0.43–6.70)	0.44
SIMV/NIV	2.32 (1.05–5.14)	0.04
Comorbidities^*^		
RDS	1.41 (0.53–3.75)	0.49
Coagulopathy	1.33 (0.53–3.29)	0.54
IVH	1.80 (0.59–5.49)	0.30

**Notes.**

Risk Ratios were obtained from Generalized Linear Models with binomial probability distribution and logit link.

* The reference group is “not present” for each comorbidity.

Abbreviations CI confidence interval C-S cesarean section EBM expressed breast milk IVH intraventricular hemorrhage NEC necrotizing enterocolitis NIV non-invasive ventilation RDS respiratory distress syndrome SIMV synchronized intermittent mandatory ventilation

In the multivariable model, NEC requiring surgical intervention was associated with an over 3-fold risk of mortality compared with medical/supportive care only (aRR 3.12, 95% CI [1.03–9.46]). The effects of birth weight, maternal history of gestational hypertension, history of antenatal steroid administration, SIMV/NIV, and comorbidity of coagulopathy, and NNJ on mortality attenuated (Table 4).

## Discussion

The proportion of NEC cases with hyperglycemia (>8.3 mmol/L) before its onset in the present study was 46.3%. We found that the use of synchronized intermittent mandatory ventilation/non-invasive ventilation was associated with an increased risk of hyperglycemia diagnosis. In addition, NEC requiring surgical intervention showed a more than three-fold risk of mortality compared with NEC treated with medical/supportive care only. The present study adds important knowledge to a relatively understudied subject in Kenya, as the most notable previous research explored the general incidence of NEC in neonates with no focus on the glycemic status (Gitau et al., 2023). However, given the small sample size and retrospective analysis, these findings should be regarded as hypothesis-generating rather than definitive.

A previous study in the UK reported a hyperglycemic rate (>8 mmol/L) of 69.3% among 95 neonates diagnosed with NEC (Hall et al., 2004). Similarly, in a Pakistan study, among those eventually diagnosed with NEC, 70.83% had hyperglycemia (Ginglen & Butki, 2025). These different incidences of hyperglycemia could be due to the varied study populations and diagnostic approaches. For instance, the UK study comprised only neonates with severe NEC admitted to the ICU, whereas the present study included both severe and non-severe cases. In contrast, the Pakistan study was a prospective research with frequent and stricter monitoring of blood glucose levels and may have detected even those with transient hyperglycemia. The present study was retrospective and relied on the data captured in the charts, which may have missed or underreported the clinically nonsignificant data. Although the proportion of patients with and without hyperglycemia was not significantly different in the present study (46.3% *vs* 53.7%, *p* = 0.39), the possible role of hyperglycemia in NEC is exemplified by Guo et al. (2020) who recently reported that perioperative blood glucose control below 8.3 mmol/L was associated with improved surgical outcomes of NEC.

Among the various factors investigated for potential association with hyperglycemia in patients with NEC, only SIMV/NIV was identified as a risk factor for hyperglycemia compared with room air ventilation. As no causative relationship between ventilation and high glycemic index has been established in the literature, the observed association could be due to confounding by disease severity. Ventilator dependence is a key indicator of disease severity, as most patients with surgical NEC develop bronchopulmonary dysplasia (Willis & Ambalavanan, 2021). Hyperglycemia has previously been associated with severe NEC (Hall et al., 2004), and hence mechanical ventilation may be a surrogate marker of NEC severity.

Although RDS was not significantly associated with the diagnosis of hyperglycemia in patients with NEC, it was the most common comorbidity overall (67.2%) and in patients with hyperglycemia (77.4%). A recent study by Berger et al. (2024) indicated that NEC was one of the adverse outcomes of RDS in pre-term infants. Patients with respiratory distress experience reduced cardiac output, and consequently, blood flow to the intestines is reduced as vital organs, such as the heart and brain, are preferentially perfused (Chandranita & Sugara, 2025). In effect, reduced blood supply to the intestines triggers ischemia-reperfusion injury of the intestinal mucosa, exposing the patients to NEC.

Despite the potential pathophysiological role of hyperglycemia in NEC, the present study showed no difference in the mortality rate between those with and without hyperglycemia. The present findings are contrary to those reported by Hall et al. (2004) who found that hyperglycemia was associated with a higher risk of mortality in patients with NEC. The inconsistent findings could be due to the different study populations, as the previous study comprised patients admitted to the ICU, with majority requiring surgical intervention for NEC. Majority of the patients in the present study had less severe NEC requiring medical/supportive care. The proportion of patients with NEC stage 3 was not significantly different between those with and without hyperglycemia. Whether hyperglycemia directly contributes to the high mortality rate or the patients’ worsening condition leads to poor regulation of blood glucose is a subject for further research (Hall et al., 2004).

The overall mortality rate of 20.9% observed in the present study is consistent with the overall mortality rate of NEC of 20–40% reported in several previous studies (Pitt et al., 2023), including 39.9% in South Africa (Selse et al., 2023), 37% in Saudi Arabia (Zhang et al., 2016), 29.8% in China (Han et al., 2022), 26.5% in the United States (Garg et al., 2021), and 21% in Poland (Gałązka, Chrzanowska & Styczyński, 2021). However, in Ethiopia, a systematic review and meta- analysis of 588 neonates reported a pooled in-hospital mortality rate of 70% (Bimerew et al., 2024). The high rate of NEC mortality rate in Ethiopia could be explained by the diverse study populations in the individual studies who were recruited from different levels of hospitals. In contrast, the present study was conducted in a tertiary and referral hospital with high-quality care, which may have led to the lower rate of NEC mortality.

The high risk of mortality in patients who underwent surgical intervention for NEC in the present study mirrors the severity of the condition. Surgery is required for severe NEC that is characterized by bowel necrosis, intestinal perforations, and sepsis. Given this advanced stage of the disease, patients have a high likelihood of a poor clinical course that progresses to mortality. In a previous study in the United States comprising 310 infants, the mortality rate of patients with NEC treated with surgery was nearly 70% (Blakely et al., 2021) against an overall rate of NEC mortality of 20–30% (Pitt et al., 2023). These findings underscore the need to monitor neonates for earlier detection of NEC and treatment, since severe NEC has poor prognosis.

Another notable finding in the present study is the high proportion of term infants with NEC (28.3%) relative to previous studies which have reported proportions below 10% (Maayan-Metzger et al., 2004; Zozaya et al., 2020). The prevalence of NEC in term infants has been reported to be increasing in recent years, but the underlying reasons are unclear. It is speculated that the presence of predisposing factors, such as tachypnea, hyperbilirubinemia, and congenital heart disease, could contribute to development of NEC in term infants (Maayan-Metzger et al., 2004). However, given the small sample size and single-center study, caution is needed when interpreting these results due to the possibility of sampling variability.

This study has some limitations. First, the retrospective design is subject to selection bias, as inclusion was limited to neonates with available and adequately documented blood glucose measurements within 72 h prior to NEC diagnosis. Infants with incomplete records or less frequent glucose monitoring may have been systematically different, potentially affecting the estimated frequency of hyperglycemia. Further, the blood glucose levels were determined through periodic measurements, but not continuous glucose monitoring. As such, clinically relevant glycemic fluctuations may have been missed in this study. Future research should employ continuous glucose monitoring to monitor the glycemic trajectories and thus ensure comprehensive measurement of the blood glucose levels.

Second, the small sample size may have reduced the statistical power of the study to detect significant association between hyperglycemia and in-hospital mortality. We performed a post-hoc power analysis, and the study yielded a power of approximately 15% for detecting an association between hyperglycemia and in-hospital mortality, indicating that the analysis was underpowered to detect modest associations. Similarly, the association observed between ventilation type (SIM/NIV *versus* room air) and hyperglycemia should similarly be interpreted cautiously given the small sample size and wide confidence intervals. These results should be interpreted as exploratory and hypothesis-generating rather than definitive. Larger, adequately powered studies are needed to confirm these findings and more robustly evaluate the association between hyperglycemia and clinical outcomes in this population.

Third, the disappearance of statistical significance in the multivariable analysis suggests potential overfitting. The number of hyperglycemia (*n* = 31) and mortality (*n* = 14) events was small and adjustment of many variables may have led to model instability. These findings warrant validation in a study with a larger sample size. Fourth, the single-center design may hinder the generalizability of the study findings to the greater population.

Despite these limitations, this study was conducted in a national tertiary hospital and included data over a 10-year period, which ensured the completeness of the data. In addition, the inclusion of all eligible neonates during the study period reduces the risk of selective inclusion, and the findings provide clinically relevant estimates that may inform hypothesis generation and the design of future prospective studies. Moreover, patient data were retrieved from both electronic and paper files, further ensuring comprehensive data capture.

## Conclusions

The present study showed that nearly half (46.3%) of the preterm and term infants with NEC were diagnosed with hyperglycemia 72 h before its onset. Hyperglycemia was not associated with in-hospital mortality of NEC cases. However, these findings should be considered hypothesis-generating, given the retrospective nature of the present study, small sample size, and single-center approach. Therefore, a prospective, large-scale, multicenter study is warranted to follow up patients with hyperglycemia for the occurrence of NEC.

##  Supplemental Information

10.7717/peerj.21601/supp-1 Supplemental Information 1 Raw Data

10.7717/peerj.21601/supp-2 Supplemental Information 2 Data dictionary

## Additional Information and Declarations

### Data Availability

The following information was supplied regarding data availability: The raw data are available in Supplemental File.
